# Immediate Effect of Neuromuscular Electrical Stimulation on Swallowing in Elderly People with Alzheimer's Dementia

**DOI:** 10.1055/s-0045-1802579

**Published:** 2025-07-25

**Authors:** Eliene Giovanna Ribeiro, Cris Magna dos Santos Oliveira, Aline Mansueto Mourão, Laélia Cristina Caseiro Vicente, Andréa Rodrigues Motta, Heitor Marques Honório, Giédre Berretin-Felix

**Affiliations:** 1 Department of Speech Therapy, Universidade Federal de Minas Gerais, Belo Horizonte, MG, Brazil; 2 Department of Speech Therapy, Faculdade de Odontologia de Bauru, Universidade de São Paulo, Bauru, SP, Brazil; 3 Department of Pediatric Dentistry, Orthodontics, and Public Health, Faculdade de Odontologia de Bauru, Universidade de São Paulo, Bauru, SP, Brazil

**Keywords:** dementia, Alzheimer disease, deglutition disorders, electrical stimulation, speech-language pathology

## Abstract

**Introduction:**

Dysphagia affects a significant number of patients with Alzheimer's dementia. Neuromuscular electrical stimulation may be a promising resource for dysphagia rehabilitation in this population.

**Objective:**

To investigate the immediate effects of neuromuscular electrical stimulation on hyoid bone displacement, pharyngeal transit time, and swallowing safety in elderly people with Alzheimer's dementia.

**Methods:**

We evaluated 30 elderly individuals with an average age of 82.79 years, regardless of the stage of dementia and with reduced hyolaryngeal elevation, using the Northwestern Dysphagia Patient Check Sheet. Neuromuscular electrical stimulation was performed at the sensory and motor levels in the submental region during videofluoroscopy, with food being offered in solid, pudding, and liquid consistencies, and in portions of 5 mL and 10 mL. We applied Analysis of variance and the Friedman test, adopting a significance level of < 5%.

**Results:**

The comparison between the sensory and motor levels of stimulation showed that there was a significant difference in hyoid bone displacement for the mushy consistency, with neuromuscular stimulation at the motor level. There was no difference in the application of stimuli for the other consistencies regarding hyoid bone displacement, pharyngeal transit time, and the penetration and aspiration scale.

**Conclusion:**

In elderly people with Alzheimer's dementia, neuromuscular electrical stimulation at the motor level generated a reduction in hyoid bone displacement during swallowing of food with pudding consistency, with no effects on pharyngeal transit time or swallowing safety.

## Introduction

Alzheimer's disease (AD) is the most common form of dementia in the elderly. Its prevalence is of 7% among individuals aged between 65 and 74 years, of 53% among those aged between 75 and 84 years, and of 40% among individuals aged ≥ 85 years. 1

Dysphagia is a frequent clinical manifestation in patients with Alzheimer's dementia, affecting approximately 32% to 45% of this population when evaluated clinically, and 84% to 93% when evaluated through instrumental examinations. 2 The signs and symptoms vary according to the stage of the disease, and they can be mainly characterized by inability to visually recognize food (visual agnosia), incoordination of tongue movements, difficulty in initiating the oral phase, significant increase in oral transit duration, and difficulty in propulsion of the bolus (which constitutes swallowing apraxia), in addition to the lack of perception of food in the mouth (orotactile agnosia). 3 4 In the more advanced stages of AD, it is common for silent aspirations to occur more frequently, which increases the risk of serious complications, such as death. 5 6

Among the protective mechanisms during swallowing, it is known that the horizontal and vertical movements of the hyoid bone during the pharyngeal phase plays a significant role in airway protection. 7 Additionally, the delayed swallowing reflex, which is common in patients with Alzheimer's dementia, can lead to an increase in pharyngeal transit time (PTT), which can also heighten the risk of laryngotracheal aspiration episodes. 8

There is difficulty in using rehabilitative techniques in patients with AD, as the individual's active participation is required, and a large number of them suffer from cognitive deficits, even in the early stages. 9 A promising modality for the rehabilitation of oropharyngeal dysphagia is neuromuscular electrical stimulation (NMES), which involves applying an electrical current to the muscles through surface electrodes in a non-invasive manner. What is different regarding this technique is its strong potential for clinical applicability in individuals unable to participate in active exercise programs. 10 11 The literature 12 13 14 presents promising results of NMES in cases of dysphagia caused by stroke, indicating positive functional changes in swallowing, increased level of oral intake, weight gain, and greater perception of swallowing capacity. Furthermore, there is efficient restoration of the movement of the hyoid bone and laryngeal elevation during swallowing, with a consequent impact on the reduction of aspiration pneumonia and hospitalizations. 12 13 14

Other studies, involving stroke and Parkinson's disease, 15 16 17 have also sought to understand the long-term effect of applying NMES to cases of dysphagia, with 10 to 30 therapy sessions lasting from 10 to 30 minutes 2 to 5 days a week . Regarding studies involving AD, only one has been found so far: in 2017, a clinical trial 18 compared traditional therapy to NMES therapy associated with electromyographic biofeedback; the authors concluded that swallowing function can be improved by the association of these approaches, but no publication on the effect of isolated NMES in individuals with AD was found.

Furthermore, in patients with advanced dementia, the literature 19 20 indicates greater effectiveness of compensatory techniques for the rehabilitation of dysphagia, as they have a positive impact on swallowing functionality by controlling the flow of the food bolus and reducing symptoms. Therefore, understanding the immediate effect of NMES and its impact on swallowing may help in the selection of stimuli, sensory or motor, to be applied safely when offering food to patients with AD before subjecting them to short-, medium-, and long-term therapies. The hypothesis of in the present study is that NMES, in the sensory and motor modalities, can cause distinct changes in the safety and temporal parameters of the pharyngeal and laryngeal functions of swallowing.

The present study aims to investigate the immediate effects of NMES on hyoid bone displacement (HBD), PTT, and swallowing safety in elderly patients with Alzheimer's dementia.

## Methods

The present is an experimental, prospective study, with a non-probabilistic sample, approved by the Ethics in Research Committee of Universidade Federal de Minas Gerais (UFMG) under number 17403613.9.0000.5149. The sample was composed of individuals who presented for evaluation at the Elderly Reference Center of the Hospital das Clínicas - UFMG.

We recruited 49 elderly individuals according to the following inclusion criteria: medical diagnosis of Alzheimer's dementia; age > 60 years; subjects without other associated neurological diseases or acute clinical conditions; ability to remain in a sitting position; full oral route; not having undergone or being scheduled to undergo speech-language pathology and audiology therapy during the study; and presence of reduced laryngeal elevation, associated or not with other changes in the pharyngeal phase of swallowing. All participants or their families signed the free and informed consent form.

After the clinical evaluation of swallowing, 13 individuals were excluded from the study for the following reasons: they did not attend or did not cooperate during the videofluoroscopic swallow study (VFSS); did not tolerate NMES; were unable to ingest the food consistencies used in the exam; or presented swallowing classified as normal when assessed by VFSS. Therefore, 30 patients were included in the study.

All patients are routinely evaluated through the Comprehensive Geriatric Assessment 21 and the Clinical Dementia Rating – CDR, 22 and all selected individuals were reevaluated by the researcher, a speech-language pathology and audiology therapist, using the Brazilian version of the Northwestern Dysphagia Patient Check Sheet. 23

### Procedures

#### Study Protocol

The instrumental swallowing assessment was performed through the VFSS, with and without the application of NMES, for liquid, pudding, and solid consistencies, in different volumes. We used a screen-printing device (model Diagnostic RX 0722, Philips, Amsterdam, Netherlands), and the images obtained in the right lateral profile, with the patient sitting, were recorded on 4.7GB DVD-R discs (Elgin S/A, Mogi das Cruzes, SP, Brazil).

**Fig. 1:**
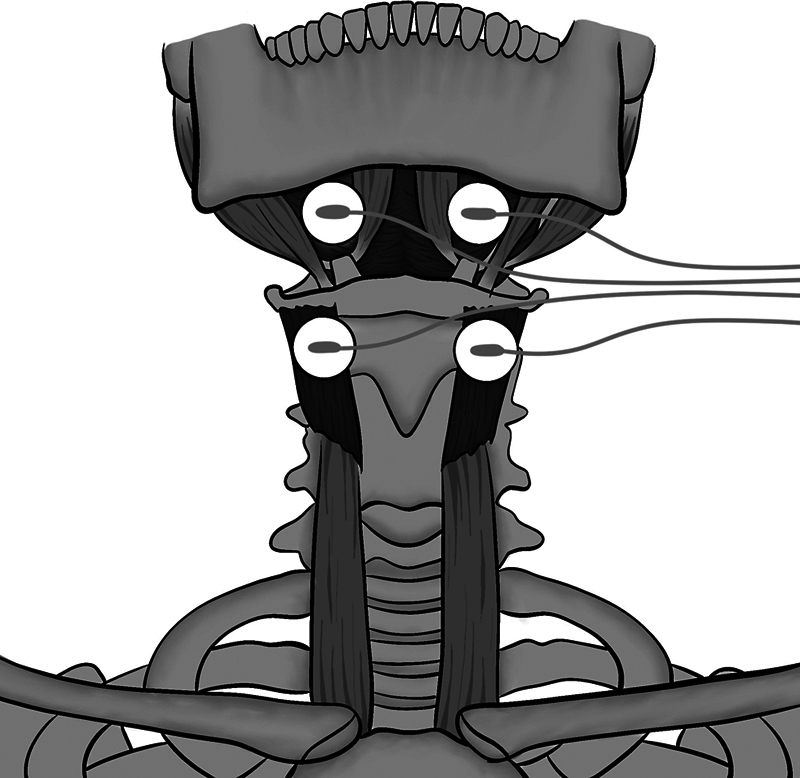
Scheme of electrode positioning for neuromuscular electrical stimulation (NMES) with one channel aligned to the region of the mylohyoid muscle and another channel aligned to the region of the thyrohyoid muscle. 24

To apply NMES, the skin on the front of the neck was cleaned with gauze soaked in 70% alcohol. Next, two pairs of electrodes were fixed ( Fig. 1 ), with one channel aligned horizontally above the hyoid bone (mylohyoid muscle region) and the second channel aligned horizontally between the hyoid bone and the thyroid cartilage, inferior and slightly medial to the posterior horn of the hyoid bone (thyrohyoid muscle region). 24 Other Brazilian studies 24 25 26 have also used the same configuration, although in different populations; therefore, due to the scarcity of investigations on the effect of NMES on AD, we decided to use the aforementioned configuration for the electrodes, even to compare the results.

#### Neuromuscular Electrical Stimulation Procedures

The NMES was performed using the portable non-invasive VitalStim therapy system (DJO, LLC, Dallas, TX, United States). The researcher, who is certified to apply NMES, used the parameters of fixed current of 80 Hz and pulse duration of 700 µs. The individual's report is normally used to determine the NMES parameters. Reports such as tingling are associated with the sensory level of electrical stimulation, and reports of clenching are associated with the motor level. Due to the prevalent cognitive deficit of the researched population, we decided to set the parameters to carry out the stimulation. Therefore, the parameters were set at 3 mA of intensity for stimulation at the sensory level and 9 mA for the motor level. 24

After positioning the electrodes, VFSS, without applying NMES, began. Then, the procedure was repeated with the application of NMES in two stimulation modalities (motor and sensory), randomly. Only individuals who showed a reduction in hyolaryngeal excursion during VFSS without NMES were invited to the next stage of the study, in this case, with stimulation at the sensory and motor levels. It should be noted that before the application of the electric current, the patient was instructed about the sensations caused by it.

When evaluating swallowing with and without NMES, the following aspects of the pharyngeal phase were observed: HBD, PTT, and presence of penetration and/or aspiration. These aspects were observed for all consistencies, which were offered randomly. Foods were evaluated in liquid, pudding, and solid consistencies, in two offers of each consistency, with a volume of 10 mL per portion of pudding food, and 5 mL and 10 mL in the liquid evaluation. For the solid consistency, we offered 1 chocolate wafer cookie, divided into 3 portions, each measuring 3 cm and containing 7.5 g. As for preparation, to achieve the pudding consistency, 3.6 g of thickener was added for every 100 mL of liquid, offered in a disposable spoon, while the liquid was offered in 50-mL disposable cups.

#### Dysphagia-Related Parameters

To characterize the sample regarding the severity of dysphagia, the Dysphagia Outcome and Severity Scale (DOSS) 27 was used. Regarding swallowing safety, the presence of penetration/aspiration was considered during VFSS, using the Penetration and Aspiration Scale. 28

The PTT was calculated for each food offered at the sensory and motor levels of NMES. For this analysis, we used markers from the video editing program Kinovea, version 0.8.15 (free and open source), which enables a frame-by-frame analysis every 3 ms. The images were used as a reference from the moment the head of the bolus reached the posterior part of the ramus of the mandible until the first frame in which the tail of the bolus passed through the upper esophageal sphincter. 29

The HBD was quantified using Image J13 software (open source). For this analysis, VFSS images, which were stopped at moments of maximum vertical movement of the hyoid bone in each consistency offered, using the Windows Movie Maker software (Microsoft Corp., Redmond, WA, United States), version 2011. For each subject, 12 images were generated (swallows of each consistency in 3 distinct moments: without NMES, with NMES at the sensory level, and with NMES at the motor level), in a total of 360 images. As a reference to analyze the elevation of the hyoid bone, we used a metallic marker positioned on the participant's mastoid region (1 cent coin).

To analyze the maximum displacement of the hyoid bone, tracings were performed on images at rest and at the moment of maximum excursion of the hyolaryngeal complex during swallowing. Initially, the intersection between a straight-line tangent to the anterior portion of the hyoid bone and another tangent to its upper margin was considered. A right angle was drawn, considering the same cervical vertebrae. The resting image was superimposed on the maximum excursion image, generating the value in centimeters, which was considered for comparison between the position of the hyoid bone at rest and in NMES.

### Statistical Analysis

To attest the reliability of the investigation methods, we conducted a concordance analysis of 20% of the sample for HBD and PTT the interclass correlation coefficient (ICC), which presented a result of 0.91 for HBD and of 0.98 for PTT. Both values are understood as an excellent level of agreement.

To verify normality, the data were subjected to the Shapiro-Wilk test. Then, repeated measures analysis of variance (ANOVA) was performed with Tukey's post-hoc test, when the data presented a normal distribution, and the equivalent non-parametric test, the Friedman Test, when there was no normal distribution. The results were reported as mean and standard deviation (SD) values for variables with a normal distribution, and as median and interquartile range (IQR) values for variables with a skewed distribution. A significance level of ≤ 5% was adopted.

## Results

The sample of the present study was composed of 30 elderly people with Alzheimer's dementia aged between 65 and 98 (mean: 82.79 ± 8.56) years and with a proportional CDR between 1 and 2. Most elderly subjects with CDR 1 were classified as having functional swallowing and mild dysphagia; those with CDR 2, with mild dysphagia; and the participants with CDR 3, with mild to moderate dysphagia ( Table 1 ), according to the DOSS. 27 .

**Table 1:** Characterization of the sample according to gender, Clinical Dementia Rating, and severity of dysphagia

Variable	N	%
Sex		
Female	22	73.3%
Male	8	26.6%
Clinical Dementia Rating		
1	12	40.0%
2	12	40.0%
3	6	20.0%
Dysphagia Severity Scale		
Moderate dysphagia	1	3.3%
Mild to moderate dysphagia	5	16.7%
Mild dysphagia	17	56.7%
Functional swallowing	7	23.3%

The distribution of the sample regarding the penetration and aspiration scale, HBD, and PTT is presented in Table 2 . Most swallows were considered normal according to the penetration and aspiration scale, both in the presence and absence of NMES. The highest mean for HBD was observed in the absence of NMES, and the lowest mean for PTT, during the sensory level of stimulation.

**Table 2:** Distribution of the sample according to the penetration and aspiration scale, hyoid bone displacement, and pharyngeal transit time

Variable	Without NMES (N = 30)	Sensory NMES (N = 30)	Motor NMES (N = 30)
Penetration and aspiration scale: (n %)			
Normal	88 (73.3%)	94 (78.3%)	94 (78.3%)
Altered	32 (26.7%)	26 (21.7%)	26 (21.7%)
Hyoid bone displacement (cm)			
Mean ± SD	2.04 ± 0.85	2.01 ± 0.78	1.91 ± 0.71
1st quartile	1.52	1.94	2.30
2nd quartile	1.50	2.00	2.32
3rd quartile	2.30	2.36	2.19
Pharyngeal transit time (s)			
Mean ± SD	1.26 ± 2.50	0.99 ± 0.86	1.15 ± 1.59
1st quartile	0.60	0.63	0.60
2nd quartile	0.70	0.76	0.98
3rd quartile	1.20	0.98	1.13

**Abbreviations:** NMES, neuromuscular electrical stimulation; SD, standard deviation.

For HBD, a significant difference was found in the statistical analysis for the pudding consistency in the group that received motor NMES compared to the group without NMES ( *p* = 0.02). As for the other consistencies, there were no statistically significant differences when comparing the NMES modalities ( Tables 3 and 4 ).

**Table 3:** Mean hyoid bone displacement regarding NMES modalities and liquid consistency

Hyoid bone displacement			
Consistency		Mean ± SD	*p* -value
**Liquid:** **5 mL**	Without NMES	2.06 ± 0.69	0.82
	Sensory NMES	2.08 ± 0.66	
	Motor NMES	2.02 ± 0.66	
**Liquid: 10 mL**	Without NMES	2.10 ± 0.76	0.11
	Sensory NMES	2.13 ± 0.86	
	Motor NMES	1.98 ± 0.73	

**Abbreviations:** NMES, neuromuscular electrical stimulation; SD, standard deviation.

**Note:** Analysis of variance (ANOVA) with normal distribution repeated measures ( *p* < 0.05).

**Table 4:** Values for hyoid bone displacement regarding NMES modalities and mushy and solid consistencies

Hyoid bone displacement					
Consistency		1st quartile	Median	3rd quartile	*p* -value
**Pudding**	Without NMES	1.39	1.88	2.32	0.02*
	Sensory NMES	1.46	1.83	2.35	
	Motor NMES	1.30	1.71	1.93	
**Solid**	Without NMES	1.60	2.12	2.44	0.19
	Sensory NMES	1.58	2.13	2.54	
	Motor NMES	1.62	1.85	2.31	

**Abbreviation:** NMES, neuromuscular electrical stimulation.

**Notes:** Friedman tests without normal distribution ( *p* < 0.05); *statistically significant.

Regarding PTT, when comparing the groups with and without NMES, there were no differences for any of the tested consistencies, with the respective values: 5 mL of liquid – *p* = 0.44; 10 mL of liquid – *p* = 0.28; pudding – *p* = 0.24; and solid – *p* = 0.07 ( Table 5 ).

**Table 5:** Values for pharyngeal transit time regarding NMES modalities and consistencies

Pharyngeal transit time					
Consistency		1st quartile	Median	3rd quartile	*p* -value
**Liquid: 5 mL**	Without NMES	0.60	0.73	0.89	0.44
	Sensory NMES	0.65	0.73	1.07	
	Motor NMES	0.60	0.72	0.87	
**Liquid: 10 mL**	Without NMES	0.60	0.71	1.10	0.28
	Sensory NMES	0.63	0.70	0.86	
	Motor NMES	0.60	0.71	0.93	
**Pudding**	Without NMES	0.59	0.76	1.42	0.24
	Sensory NMES	0.60	0.77	1.37	
	Motor NMES	0.60	0.71	1.33	
**Solid**	Without NMES	0.61	0.71	1.90	0.07
	Sensory NMES	0.57	0.80	1.31	
	Motor NMES	0.73	0.93	1.43	

**Abbreviation:** NMES, neuromuscular electrical stimulation.

**Note:** Friedman tests without normal distribution ( *p* < 0.05).

As for the penetration and aspiration scale, the statistical analysis showed that, when comparing the groups, there were no significant differences: 5 mL of liquid – *p* = 0.54; 10 mL of liquid – *p* = 0.46; pudding – *p* = 0.54; and solid – *p* = 0.89 ( Table 6 ).

**Table 6:** Values for the penetration and aspiration scale regarding NMES modalities and consistencies

Penetration and aspiration					
Consistency		1st quartile	Median	3rd quartile	*p* -value
**Liquid: 5 mL**	Without NMES	1.00	1.00	1.00	0.54
	Sensory NMES	1.00	1.00	1.00	
	Motor NMES	1.00	1.00	1.00	
**Liquid: 10 mL**	Without NMES	1.00	1.00	2.00	0.46
	Sensory NMES	1.00	1.00	2.00	
	Motor NMES	1.00	1.00	2.00	
**Pudding**	Without NMES	1.00	1.00	1.00	0.54
	Sensory NMES	1.00	1.00	1.00	
	Motor NMES	1.00	1.00	1.00	
**Solid**	Without NMES	1.00	1.00	1.00	0.89
	Sensory NMES	1.00	1.00	1.00	
	Motor NMES	1.00	1.00	1.00	

**Abbreviation:** NMES, neuromuscular electrical stimulation.

**Note:** Friedman tests without normal distribution ( *p* < 0.05)

## Discussion

Swallowing impairment is expected in the intermediate and late stages of AD; however, recent studies 3 30 have reported the presence of altered function since the initial stages. This was observed in the results of the present study, revealing that CDR 1 individuals already had some degree of dysphagia, measured by the DOSS, with worsening according to the severity of the disease. 25 The present study showed that the motor stimulus of the NMES resulted in a decrease in HBD during the evaluation of the mushy consistency. However, no significant differences were found in PTT or on the penetration and aspiration scale between the groups with and without NMES.

To date, the present is the first study carried out with the aim of investigating the immediate effects of NMES in AD to understand the safety of using this therapeutic approach in the long-term treatment. The immediate effect is the changes observed during the application of electrical stimuli, with a single moment of application and evaluation during the VFSS examination. In the literature, we only found one study, 18 published in 2017, that sought to understand the effects of NMES associated with electromyography on swallowing in patients with AD. The authors 18 evaluated a sample of 103 participants divided into 2 groups, one that received traditional therapy for dysphagia and the other who received NMES therapy combined with electromyography for 12 weeks, and they concluded that the combined therapy was more effective in improving swallowing safety and reducing the severity of dysphagia.

Regarding the findings of the current study, we highlight that the reduction in HBD may be justified by the action of electrical stimulation, which maintains some degree of contraction of the supra- and infrahyoid muscles, generating greater resistance to hyolaryngeal elevation during swallowing. 31 The elevation of the hyoid bone is essential for airway protection, as it aids in closing the airway and prevents penetration and aspiration by moving the larynx upward and forward, which facilitates the closure of the epiglottis over the airway entrance. 32 However, the sample of the present study was composed of elderly people, and it is known that HBD may decrease with aging, possibly as a compensatory strategy for physiological changes, 32 such as a lower laryngeal position, which may be an adjustment that leads to a decrease in protection of the lower airways.

Training of swallowing with effort, associated with electrical stimulation, was demonstrated in a 2009 study 15 with healthy individuals; the authors concluded that there is an increase in elevation of the hyoid with this type of task. Still investigating the effects of NMES in healthy people, another study 33 also showed that there was a decrease in the elevation of the hyoid with the motor level of stimulation (in this case, only the swallowing of liquid consistency was evaluated). In another study, 34 the reduction in HBD with motor NMES was associated with a decrease in penetration/aspiration, which, according to the authors, may be related to the effort that the patient needed to make to elevate the hyolaryngeal complex during swallowing. This same study 34 discusses that this effect of HBD reduction seems to contribute to the individual involuntarily swallowing with effort, with this movement having a positive contribution to the muscles involved in swallowing. 31 Thus, according to the results of the present study, we hypothesize that the use of NMES may assist in swallowing with greater effort due to resistance to hyolaryngeal elevation with stimulation. This may be an alternative to induce increased laryngeal elevation and consequently work on the safety of passive swallowing, considering individuals with AD who do not undergo active therapy.

In the analysis using the scale by Rosenbek et al., 28 most participants of the current study were classified within the penetration levels, without the occurrence of aspiration. Furthermore, there was no negative effect of NMES on swallowing safety among the participants, which demonstrates that this type of electrical stimulation does not put the patient at risk. It is worth highlighting that the sample herein analyzed was in the initial stage of AD, that is, with a lower probability of penetration and aspiration episodes. We suggest that individuals with a history of changes in swallowing safety be evaluated in future studies, so the effect of NMES in this aspect can be better understood.

Regarding PTT, as well as the findings of the current study, a group of North American researchers 33 evaluated the effect of motor NMES on liquid swallowing (in this case, in healthy individuals), and they detected no influence of electrical stimulation on this parameter. In this context, some authors 33 34 hypothesize that the reduction in HBD by motor NMES may result in resistance to the upward movement of the tongue during swallowing, causing a decrease in the pressure at the base of the tongue and, consequently, in PTT. Although the present study does not confirm this hypothesis, it is possible that electrical stimulation, both motor and sensory, is a promising approach for individuals with AD, especially for those patients who are no longer able to perform active exercises. In healthy people, sensory NMES showed a tendency to increase pressure at the base of the tongue; 24 however, new clinical trials are needed to understand whether this effect would influence PTT.

Regarding the parameters to be used for the application of NMES at the sensory and motor levels, no data on ideal and recommended values were found in the literature. In our case, the use of fixed application parameters 24 of NMES had healthy elderly people as a reference, based on patients' signaling of sensory definition and muscle contraction thresholds. Thus, in future studies, it is necessary to establish the parameters of NMES for this specific population.

Furthermore, even for statistical purposes, the sample should be considered relatively small, but compatible with those of other studies 14 35 36 , which have evaluated 10 to 40 participants. In addition, we propose an investigation on the use of NMES in long-term therapies in individuals with AD, based on the finding of the current study that, from the moment in which no losses were observed in the physiological aspects studied, it becomes safer to continue with the deepening of therapies with longer stimulus time. Additionally, a limitation of the present study was that patients in more severe clinical conditions were excluded from the sample due to the requirements defined for the study. Therefore, it is necessary to include this patient profile in future research to gain a deeper understanding of the effects of NMES.

The importance of developing new research with this aim is also supported by the fact that NMES can be understood as an important and valuable tool for speech-language pathology, as it promotes reorganization of the cerebral cortex through an increase in sensory input, with sensory stimulation, and activation of muscle fibers, with motor stimulation. 36

## Conclusion

The application of NMES did not generate immediate changes in PTT and swallowing safety in elderly people with AD. Furthermore, stimulation at the motor level reduced HBD during the swallowing of the mushy consistency.

There is a need for additional studies with individuals with AD in more advanced stages to better understand the influence of NMES on the physiological aspects of swallowing.
